# Exploring Gameful Motivation of Autonomous Learners

**DOI:** 10.3389/fpsyg.2022.825840

**Published:** 2022-02-25

**Authors:** Jukka Vahlo, Kai Tuuri, Tanja Välisalo

**Affiliations:** ^1^Department of Music, Art and Culture Studies, University of Jyväskylä, Jyväskylä, Finland; ^2^School of Economics, Centre for Collaborative Research, University of Turku, Turku, Finland

**Keywords:** playfulness, games, autonomous learner, online learning, motives, motivation, engagement, self-determination

## Abstract

In this explorative study, we investigated motives of autonomous learners to participate in an online course, and how these motives are related to gameplay motivations, engagement in the course experience, and learning outcomes. The guiding premise for the study has been the idea that learning and game playing carry phenomenal similarities that could be revealed by scrutinizing motives for participating in a massive open online course that does not involve any intentionally game-like features. The research was conducted by analyzing survey data (*N* = 705) collected from individuals who had voluntarily participated in an open online course about artificial intelligence and its societal impact. The survey included an explorative Motives for Autonomous Learning (MAL) inventory. Exploratory factor analysis suggested that the MAL inventory consisted of six dimensions out of which four were consistent with factors that earlier research has associated with motives to engage with video games. Of the identified factors, the dimension that most clearly described autonomous and playful predispositions was found to be a main precedent for both experienced gamefulness of the learning experience and positive learning outcomes. In all, the results of this study demonstrated that playfulness and autonomy were both prominent and significant factors across the whole learning process.

## Introduction

People all around the world spend an astonishing number of hours playing video games each day. Today, this is not only true for young men but rather for people representing all genders and ages as players’ mean age is already close to 40 years (Vahlo et al., 2018; Kinnunen et al., 2020). Consequently, both researchers and game industry representatives are keen to understand what motivates players to begin to play and what keeps them engaged with the same application often for hundreds of hours or even more (e.g., Ryan et al., 2006; Sherry et al., 2006; Yee, 2006; Przybylski et al., 2010; Yee et al., 2012; Kahn et al., 2015; De Grove et al., 2017; Vahlo and Hamari, 2019). Indeed, the literature on player motives is a rapidly growing area of research and many models have been published and empirically validated during the past two decades. In the field of education research, the tradition of studying learners’ motivation extends back even further in history. The studies of learning motivation revolve around questions very similar to the ones in game motivation research, that is, the general aim is to understand what ignites the interest for learning and what keeps learners engaged with their studies (e.g., Skinner and Belmont, 1993; Appleton et al., 2006; Zepke and Leach, 2010; Christenson et al., 2012; Sinatra et al., 2015).

The relationship between play and learning has been acknowledged and also studied extensively since the early 20th century (Curtis, 1915; Coleman, 1971; Bruner et al., 1976; Vygotsky, 2004). The emergence of digital gaming further increased researchers’ interest in games for learning (e.g., Gee, 2003; Squire, 2003). As a consequence, serious game (i.e., edutainment) applications (e.g., Charsky, 2010) and learning applications with gamified elements (e.g., Sailer and Homner, 2020; Zainuddin et al., 2020) have established themselves as common practices in their respective fields. Yet, there is much that is still not understood about the relationship between learning motivation and motivation to play video games. This area of investigation has a potentially high research impact and societal implications, as being motivated is known to be associated with higher levels of engagement with learning activities and positive learning outcomes (e.g., Janosz, 2012).

While there is a long tradition in investigating learning motivations and while literature on game playing motivations is expanding rapidly, there exists a surprisingly limited number of studies on how gameplay motivation might be interwoven with *learning motives*. There is an evident need for understanding these interconnections as many learning experiences of today take place in digital environments that could make use of game-based solutions in a profound and versatile fashion. And there is even more prominent justification for setting the focus specifically on learning motives (e.g., different reasons and other predispositions for learning). The reason is that motives for learning are not that often made explicitly visible in the motivation research models used in the field of education, apart from more informal learning perspectives concerning professional or higher education (see, e.g., Bahn, 2007; Diseth et al., 2010). Within the contexts of more traditional or formal education, the models utilized for studying learning motivation (see, e.g., Skinner and Pitzer, 2012; Virtanen et al., 2014) tend to underline the teachers’ and school’s ability to support engagement with learning activities, teachers and peers rather than individual predispositions (i.e., motives) for voluntarily choosing what to learn.

Engagement with game activities is a central subject for game motivation research as well, and even the concept of playfulness (and playful motivation) is considered as an experiential property that gets (re)organized in engaging with contextual activities (Masek and Stenros, 2021). The viewpoint of game research, however, appears to have a somewhat different basis in approaching the voluntary nature of the activity. Throughout the existence of game research as a field of academic inquiry, it has emphasized that player participation in game experiences is characteristically *voluntary* and *autonomous* (Huizinga, 1949; Goffman, 1959; Goffman, 1961; Suits, 1978; Caillois, 2001). The models of game motivation research typically build upon this premise and often emphasize the individual’s motives for playing (e.g., Sherry et al., 2006; Yee, 2006; Vahlo and Hamari, 2019). In exploring linkages between gameplay and learning motives, we consider it necessary to apply a similar framework of autonomous and voluntary participation as a basis of our inquiry. It is crucial to note that much of the learning motivation research assumes the existence of a formal institutional context (e.g., a classroom setting in a school) and a generalized achievement structure (e.g., curriculum and the related degrees) within which the motivations for learning activities are developed. With respect to a growing number of today’s online learning services and online courses, however, it is evidently not valid to assume such circumstances that contribute to the learner’s motivational disposition. Furthermore, also in formal institutional contexts, more consideration should be given to understanding how learners’ motives to participate may impact their learning experience and also learning outcomes. In this paper, we have chosen to emphasize the viewpoint of an *autonomous learner*, which embraces the free will of a learner to reflect on the reasons for their participation and even choose whether or not to participate in a course in the first place. This approach puts motives into the spotlight, and it is especially relevant to the different forms of adult education and massive open online courses (MOOCs), where participation in learning activities is usually more or less voluntarily initiated.

In this explorative study, we empirically investigate the motives of autonomous learners to participate in an online course and how these motives are related to gaming motivations, course experience and engagement, and learning outcomes. This will be done by analyzing survey data (*N* = 705) collected from individuals who voluntarily participated in an online course, “Elements of AI,” about artificial intelligence and its societal impact. By studying course participants’ experiences, we explore how playfulness should be understood both from the perspectives of an autonomous learner (i.e., playful motives) and the learning situation (i.e., playful engagement with learning). In general, our aim is to unveil the playfulness inherent in learning experiences, which do not include any intentionally game-like design solutions, and how this playfulness associates with learning motivation and learning outcomes.

## Theoretical Background

### Combining Educational and Gaming Contexts

Utilization of games as technologies of learning and pedagogical tools has a long history that precedes the current era of digital gaming (Coleman, 1971). Likewise, the study of play in education and human development has been established decades ago (e.g., Groos, 1908; Piaget, 1952; Bruner et al., 1976; Vygotsky, 2004). Being playful is associated with spontaneous learning in seemingly aimless activities (Lieberman, 1977). It is widely acknowledged, also within the field of game design (e.g., Crawford, 1984), that gameplay in itself incorporates learning processes, even when games are not deliberately meant to teach us anything outside of how to play the game itself. Games have also been criticized for teaching the subject matter through an inherently mechanical and reductionist simulation (Kraft, 1971), but on the other hand, the very ability of providing predictable simulated realities, in which a learner can experience agency while engaging with the playing, is considered to be an intrinsic virtue of games in education (Coleman, 1971). In this paper, the main rationale for combining educational and gaming contexts in studying motivation lies in two premises: (1) motivational factors for playing games potentially overlap with the motivational factors for engaging oneself voluntarily and autonomously in learning, and (2) all learning experiences necessarily include elements that can be considered to be *gameful* as these elements are necessary for all game playing experiences.

The modern conceptualization of digital games tends to separate the domains of entertainment and serious gaming. The educational functions of digital games are thus often seen only in the light of the serious and purposeful activities that aim for learning outcomes, in contrast with “just playing for fun.” Such a distinction between “fun” and “serious” games is problematic in many respects, and it is not necessarily justified when playful learning processes and gameful student engagement are concerned. From the perspectives of evolution and human development, play literally is a “serious” practice and an important adaptation method as well as a method for learning skills that are ultimately essential for survival. On the other hand, gameplay could be characterized as a safe constitution of “reality” (e.g., Piaget, 1952; Stenros, 2014) and as an activity that captivates its actors, induces passion, and facilitates social bonding (e.g., Whitton and Moseley, 2014). In all its seriousness, gameplay thus may be absorbing, engaging, and, in all, an entertaining experience regardless of whether the game has been designed with a particular learning purpose in mind.

The most prominent justification for game-based learning (Prensky, 2001) or gamification of learning (Deterding et al., 2011; Faiella and Ricciardi, 2015) has been that games are effective in motivating and engaging students in learning activities. This effect has been found to be similarly important for other kinds of serious games, such as games for health (Kowert and Quandt, 2016). By definition, games include conditions that players need to meet in order to progress in the game. These conditions are environmental demands that impose challenges for the player’s performance. An optimal gameplay experience is often depicted as the player acting in a state of flow, where there is a balance between the challenges of the game and the player’s abilities. It has been shown that, within such approaches, this type of engagement indeed has a positive effect on learning (Hamari et al., 2016). According to Mayer’s (2019) review, previous research on game-based learning has focused on (1) the important features in games that support learning, (2) the potential learning effects of commercial video games, and (3) the benefits for learning through games when compared to traditional teaching methods. Although there is increasing knowledge on the educational benefits of computer games (Mayer, 2019), the gameful features, such as challenge and flow (Hamari et al., 2016), that work for learning, as well as instructional techniques of serious games, such as context integration, narration-based techniques, feedback, and adaptivity (Wouters and Van Oostendorp, 2017), it is still not well understood in which situations these features work the best, and for who, what are the underlying motivational cognitive, affective and social mechanisms explaining these effects (Faiella and Ricciardi, 2015; Mayer, 2019).

Instead of focusing on the ways games work as motivators for learning, our focus is on discovering how motives to participate in an online course are potentially associated with the perceived playfulness/gamefulness of a learning experience which does not include a standalone game and which has not even been purposefully gamified. In addition, we are also interested in understanding how both motives to participate and gameful qualities inherent to the learning experience (as a part of the given motivational climate) are associated with learning outcomes.

Our scrutiny aims to treat both contexts on an equal basis: play is viewed as a natural part of knowledge and skills creation, and playfulness as a general motivational foundation in humans (e.g., Masek and Stenros, 2021). At the same time, we presume that learning inevitably incorporates aspects of play and game-like activities. Therefore, for game players and online learners alike, it makes sense to equally ask questions related to their “gameful” and “learnful” motivations. In the present study, our focus is set on online learners but the integrative strategy with respect to the two motivational contexts remains the same.

### Autonomy of a Player and a Learner

Motivation to play games is often conceived through intrinsic motivation, underlining the spontaneous curiosity, interest, and free will of a player (Ryan and Deci, 2000; Sansone and Harackiewicz, 2000; Rheinberg and Engeser, 2018). Unlike the purposeful activities that we do in order to provide sustenance for ourselves and our community, play does not seem to have any obvious goal. Therefore it seems to epitomize humans’ desire to act on a purely voluntary basis—basically just for fun. This is not to say, however, that play would not have any benefits. Human play is associated with physical and psychological wellbeing (Proyer, 2013; Kowert and Quandt, 2016) and has various functions that relate to physical activity, exploration with objects, and socially valuable pretense behavior (Sutton-Smith, 1983; Smith, 2005). From the developmental and evolutionary perspective, play and games have functioned as pivotal ways of learning (e.g., Bruner et al., 1976). For a child, playing might be just a fun activity, but implicitly children also develop their physical or social skills that are relevant and useful in their future life (Piaget, 1952). It has even been argued that abilities to play and imagine have been key preconditions in the survival and development of the human species (Huizinga, 1949). From this perspective, it is intriguing to consider play as a human need for actualizing one’s playful self (i.e., *homo ludens*). Presumably such a need is strongly tied to autonomy—a “capacity for and desire to experience self-regulation and integrity” (Deci and Ryan, 2012, p. 85). Because of the close relationship of play and learning, it is equally tempting to consider that there could be some profound similarities between an autonomous player and an autonomous learner.

Nevertheless, games, as a form of organized play, can be approached from a seemingly contradictory direction, as the rules and goals they impose on a player can also be described as external control systems (Avedon and Sutton-Smith, 1971, p. 7), which effectively regulate the player’s motivation and action. The procedural control is maintained through either persuasive or intrusive rhetorics and contribution to the player’s motivational self-regulation (Bogost, 2007). This kind of understanding of player participation assumes that a player’s desire to play is *given* and that the player is not a fully free subject who can make decisions as she chooses (Aarseth, 2007). Instead of being a free subject, the player is subjected to the *gameplay condition*, that is, she is a player only because she agrees to fulfill the requirements the game sets for their interaction (Leino, 2010). However, in the enactive approach to human cognition it is conversely argued that an environment does not impose any dictating condition over an autonomous subject (Weber and Varela, 2002; Thompson, 2007). Instead, it is the individual, and in this context a player, who voluntarily meta-regulates her ongoing playful player–game interaction (Vahlo, 2017). Games represent a type of environment, which may obviously incorporate persuasive design features, such as rewarding possibilities for action (Hamari et al., 2014), but that does not mean that a player necessarily experiences any external control as long as it is coherent with her self-regulative orientation in engaging with the play-world (see Tuuri et al., 2017). A person’s intention to play, her motives, and her playful dispositions are all essential constituents of the game experience (Sutton-Smith, 2009; Karhulahti, 2015). In terms of comparing the autonomies of a learner and a player, learning activities too are often externally (i.e., pedagogically) organized, in one form or another. In the present study, our focus is on an open online course, which is voluntarily chosen by its participants—just as games are voluntarily chosen by their players.

### Ecological Approach to Motivational Development: Taking Contextual Engagement Into Account

Our understanding of motives and motivations is based on how the human mind is conceptualized in ecological and enactive approaches to psychology. The basic premise in these approaches holds that the human mind is not just inside the head but extends to everything that the head and the body are inside of (Gibson, 1977; Mace, 1977; Noë, 2009). This demarcation states that the organization of our perception, action, and knowing—basically also the substrate of motivational development—relies on embodied interactions that couple our cognition with the environment (see also Varela et al., 1992). Autonomy, and especially biological autonomy, has been described as a core tenet of enactive approaches to cognition (Di Paolo et al., 2010; Froese and Stewart, 2012). The concept of autonomy is defined as a core organizing principle for all living systems, and it is directly associated with the identity and survival of an individual who is required, hence motivated, to interact with the world to maintain its autonomy (Thompson and Stapleton, 2009; Di Paolo and Thompson, 2014).

Of the broader field of research on human motivations, a specific interest of this study concerns *explicit motives*, which denote certain “predispositions to approach a particular class of incentives or to avoid a particular class of threats” (Thrash et al., 2012, p. 141). Differently put, explicit motives are reasons individuals hold for their involvement in particular activities, and therefore, these motives are also self-attributed conscious expressions of individuals’ values, goals, and self-identities. Explicit motives have a directive function in our decision-making and reasoning whereas unconscious *implicit motives* serve as an energizing function in our actions (McClelland et al., 1989; Thrash et al., 2012; Brunstein, 2018). The approach of explicit motives is widely utilized in self-report surveys and questionnaires in which respondents operate with verbally encoded explicit motives (Thrash et al., 2012). In these studies, explicit motives can be treated as verbalized results of a respondent’s self-reflective cognitive appraisal of the mentioned predispositions to act on the environment.

Explicit and implicit motives are useful concepts in studies which aim to understand how individuals make sense of their own decision-making and how motives which they consider to be important predict patterns of their behavior as well as outcomes of their actions. However, within a broader theoretical model, motives may appear as static reflections of an essentially dynamic motivational development. Behind motives, we can outline several different motivational dispositions that are potentially involved and projected in the constitution of a motive. Such motivation background can consist of, for example, personal goal pursuit and the related commitment (Gollwitzer and Oettingen, 2012), achievement goal orientation (Elliot and Thrash, 2001), social self-regulation (Schunk and Usher, 2012), playful attitude/intentions (Masek and Stenros, 2021), culture and personality (Higgins, 2008), or self-regulation in terms of basic need satisfaction and one’s autonomic “true self” (Deci and Ryan, 1991). Explicit motives to participate, for instance, in an online course, potentially incorporate a contextual and situated foundation of different background elements. While the motivational background arguably contributes to the motive generation, in a simple verbalization of an explicit motive, it may remain only implied.

Explicit motives are products of making sense of a person’s motivational predisposition for activity. But they should not be taken merely as propositional reasonings or planning that precedes action. According to the framework of the embodied and ecological mind, human motivation and the appraisal of explicit motives are necessarily co-constituted in engagement with a given environment. Similarly to Gibson’s (1977) concept of action affordances, motivational cognition is neither a property of a living entity nor a property of an environment, but rather both of them, being enactively structured (Varela et al., 1992) through interactions with the environment. The essence of motives (as predispositions to approach or avoid) are thus not detached from a person’s experiential history of activities and contexts they relate to, including what kind of outcome expectations an individual has for participating in these contextual activities (Schunk and Usher, 2012).

**Figure 1:**
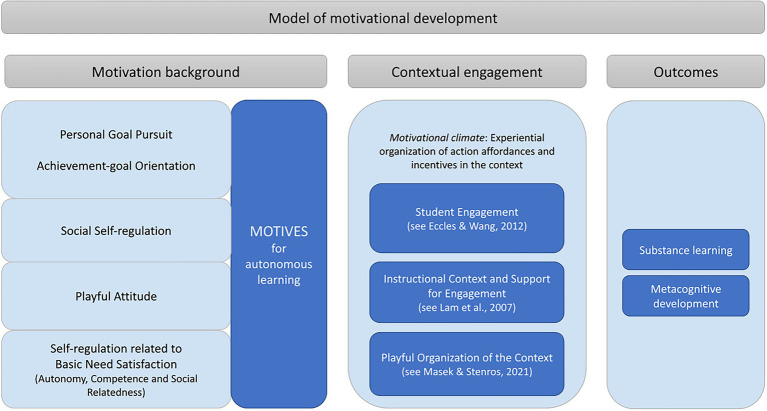
The model of motivational development consisting of three domains (motivation background, contextual engagement, learning outcomes) and their interrelations.

All this is to say that motives are best scrutinized in dynamic connection with the contextual processes of motivational development and *engagement*. Similarly to Skinner and Pitzer’s (2012) model of motivational dynamics in learning, we see contextual engagement with (learning/gaming) activities pivotal both in understanding the adaptive and self-regulative processes of motivation, being tied in situational engagement, and embedded in the *motivational climate* that is afforded to the actor by the context (see Figure 1). Motivational climate is here broadly understood as the overall motivational “environment” that contributes to the experiential organization of contextual action affordances and incentives, instructional elements, autonomy and control, communal support, and consequently to the ways how engagement with activities is structured. As contextual engagement and motivation background are interconnected, the climate arguably also contributes to the development of individuals’ motives and motivational orientations (such as mastery or performance approaches, see Ames, 1992, p. 262). Unlike the model of Skinner and Pitzer, however, the model of motivational development constructed for the use of this study does not presuppose any context of activity, such as classroom, school, or any other institutional establishment.

Masek and Stenros (2021) have recently conducted a systematic, integrative literature review on the concept of playfulness across different scientific disciplines. According to the review, conceptual usage of playfulness in the literature is essentially two-fold: it is either taken as an *individual’s desire or willingness for playful engagement* in relation to context(s), or it is understood in terms of *playful organization of the context*. This conceptual synthesis makes it clearer how we position playfulness or gamefulness into a model of motivational dynamics. Correspondingly, playfulness can potentially be considered both as (1) background intent for (playful and gameful) explicit and implicit motive generation, and (2) structural organization of (playful and gameful) experience of engaging with activities in the context. Conforming to this idea, our model of motivational development (see Figure 1) incorporates playfulness both in a person’s dispositional motivation background (i.e., playfulness implied in motives) and contextual engagement (i.e., experientially playful organization of the context). Again, we assume that both the individual’s playful disposition and the contextual engagement of playfulness are interconnected. For example, if a person has a motive that reflects “a desire for playful engagement” it should be likely that the playfulness will also be embodied in the experiential organization of the context.

### Research Questions and Hypotheses

Our survey targeted autonomous learners who participated in an online course on artificial intelligence and its societal impacts. Conforming to the model of motivational development presented above, the research focuses on the following three phases of the learning process: (1) motives to participate in the course, (2) contextual engagement during the course, and (3) learning outcomes. In line with this demarcation, the research questions of this study are: (1) How do playful and gameful intent for motive generation and for structural organization of a learning experience manifest themselves in self-report data about users’ motives to participate in an online course; (2) how are aspects of playful and gameful intent in motive generation related to playful and gameful organization of learning experiences in the engagement with the course; and (3) how are playful/gameful motives (the first item) and playful/gameful organization of experiences (the second item) associated with learning outcomes?

Consequently, the hypotheses of this study are as follows. First of all, because of the potential overlap between learning intents and a desire for playful engagement, we expect that this study will unveil structures in the motives of autonomous learners that resemble motives for playing games, as both of these modes of participation are profoundly voluntary and interactive.

*H1: Course participants’ explicit motives to participate in an online learning course reflect their intent for learning but also resemble prevalent motives to play digital games.*

Moreover, we expect to find gameful qualities in the experiences of situational engagement with learning activities. This is because learning activities share phenomenal qualities with game activities as both impose interactive tasks, challenges, goals, and performance evaluation (Lam et al., 2007; Adams, 2014; Karhulahti, 2015). Furthermore, gameful qualities of a situation are co-constituted both by the environment and the individual’s ways to organize the situation in a playful manner.

*H2: Gameful qualities are not exclusive to games and gamified solutions but gameful qualities are also manifested in those learning situations and activities that are not strictly game-based or intentionally gamified.*

Our third hypothesis concerns the relationship between motives and engagement. As there seems to be an agreement in the field of educational research that “motivation is a basis for subsequent engagement” (Martin, 2012, p. 305), we can expect a positive association between the two. Furthermore, we expect that the experienced gameful qualities parallel the experience of engagement with learning activities. This is because gameful experiences arguably presuppose active involvement (i.e., engagement) by the learner (Högberg et al., 2019).

*H3a: Identified motive dimensions (H1) all predict a higher learner engagement with the online learning course.*

*H3b: Experienced gameful qualities (H2) of a learning situation are associated with learner engagement.*

Since the gameful qualities of a situation are co-constituted by both the learning environment and the individual’s intent to organize the situation in a playful manner, we assume that learners’ playful motives contribute to the perception of gamefulness of learning. Individuals’ motives to participate are not to be understood as isolated from the ongoing experience, but rather as a meta-regulative resource they utilize before, throughout, and after their participation (see also discussion on engagement process in Janosz, 2012, p. 699).

*H4: Experienced gameful qualities of the online course are not the same for all participants but are influenced by their motivation for organizing the learning situation playfully. Therefore, the explicit motives that participants hold predict how they experience the gamefulness of the learning situation.*

Our final hypothesis considers the experienced learning outcomes, and how these associate with motives and engagement. Previous research (see Janosz, 2012) points out not only that motives are determinants of learning engagement, but also that engagement has an effect on student learning success. Therefore, we expect the following:

*H5a: Contextual engagement is a precedent for learning outcomes.*

*H5b: Explicit motives are all associated with learning outcomes via contextual engagement.*

## Materials and Methods

### The MAL Inventory Development

Research data was gathered using a survey targeted at students of Elements of AI, a MOOC developed by the University of Helsinki and the software company Reaktor. The course includes learning materials and assignments and is not purposefully gamified. For measuring explicit motives of participating in the course, the survey included an explorative Motives for Autonomous Learning (MAL) inventory. Since explicit motives are conceptualized to be relatively stable dispositions and “reasons people hold for initiating and performing voluntary behavior” (Reiss, 2004), they are considered well-suited for survey-based research setups (Beckmann and Heckhausen, 2018; Scheffer and Heckhausen, 2018; Schultheiss and Wirth, 2018). Motive questionnaires assess the self-attributed explicit schemata of individuals that direct and select their behavior, usually across situations (Thrash et al., 2012, pp. 133–134).

In the scale development process, we followed a five-step procedure presented by Phan et al. (2016). These steps consist of preliminary item pool generation, expert review of the item pool, a questionnaire pilot study, preferably two exploratory factor analyses (EFAs), and a theory-based confirmatory factor analysis (CFA) including its validity and reliability testing. Since the objective of this explorative study was to examine the dimensionality of the motives for the autonomous learning inventory and how these motives are associated with learning experiences and outcomes, our scale development did not include confirmatory phases for the MAL scale development. Psychometric validation of a scale necessitates at least two EFAs and a CFA with different survey data (Matsunaga, 2010), and both of these steps fell beyond the scope of the current study. As background material for designing a preliminary item pool for MAL, we used open-ended data originally collected by Reaktor about the students’ reasons for participation. These responses (*n* = 225) were first coded into potential motive categories based on recurring themes in the individual responses and a total of 34 motive categories were identified. The 34 categories were further classified into nine main categories: competence, mood management, achievement, social, boredom, autonomy, fun/entertainment, curiosity/interest, and fear of missing out. Both the 34 categories and the nine main categories were reviewed by three researchers and two industry operators as a part of the expert review phase of the scale development.

Next, the researchers went back to the data and selected those motive descriptions that captured different aspects of the nine main categories and the 34 sub-categories. However, not all nine preliminary motive categories were equally represented in the open-ended data. In order to balance the inventory and to construct new survey items for the motive categories, we reviewed a series of motive inventories that assess reasons to play digital games (Sherry et al., 2006; Yee, 2006; Demetrovics et al., 2011; Yee et al., 2012; Kahn et al., 2015; De Grove et al., 2017; Vahlo and Hamari, 2019). As a result, we constructed MAL inventory items (Table 1) for each of the preliminary nine categories as our objective was to include the preliminary MAL inventory in a questionnaire pilot study.

**Table 1:** The 35-MAL Inventory and its descriptive statistics.

*N* = 705	Description	Mean	SD
item1	To get the credits	3.01	1.99
item2	Because of the certificate	3.32	1.89
item3	To build up my resume	3.61	1.92
item4	To catch my dream	3.01	1.66
item5	Because it was free	5.48	1.56
item6	Because I could study anytime and anywhere	5.72	1.29
item7	Because it opens up future possibilities	4.49	1.66
item8	Because I was bored	2.29	1.57
item9	Because I had too much free time	2.41	1.70
item10	Because I had nothing else to do	2.35	1.59
item11	To use it in my profession	4.02	1.93
item12	To build up my skills	5.90	1.09
item13	Because of the challenge	4.89	1.67
item14	To learn something new and useful	5.93	1.02
item15	To explore new domains of knowledge	5.78	1.13
item16	To contribute to my creative thinking	5.11	1.55
item17	Out of curiosity about what the future might bring	5.96	1.08
item18	Because I am interested in AI	5.97	1.10
item19	To keep up with the times	5.63	1.21
item20	Because of the fear of missing out	2.97	1.70
item21	To stay ahead of technological disruptions	5.60	1.26
item22	To stay current with innovations	5.70	1.18
item23	For fun	5.12	1.54
item24	Because of entertainment	4.08	1.84
item25	Because it made me feel good about myself	4.76	1.66
item26	To overcome the fear of AI and robots	1.99	1.45
item27	To speak about the subject intelligently	4.53	1.66
item28	Because it was recommended to me	3.28	2.00
item29	Because my friends or colleagues took the course	2.47	1.76
item30	Because everyone needs to know about this subject	5.27	1.50
item31	Because I was encouraged to take the course	2.88	1.85
item32	I was curious about the subject matter	6.10	0.95
item33	To use the understanding of the subject in my studies or in my job	3.67	1.99
item34	To advance my career	3.52	1.83
item35	Because I wanted to evaluate the course quality	2.97	1.83

Before an EFA was made for the MAL inventory to investigate latent motive categories of the autonomous learners, we assessed the sampling adequacy of the 35-item inventory. The inventory passed the Kaiser–Myer–Olkin (KMO) test with a value of 0.88 and also the Bartlett test of sphericity (*chi-square* = 9,673, *df* = 595, *p* = 0.000), both of which indicated that a factor analysis was appropriate.

### Measuring Engagement, Experience, and Outcomes

GAMEFULQUEST is an instrument for assessing the perceived gamefulness of using any service or system (Högberg et al., 2019). In the context of our study, “the service” refers to user experience when participating in an online learning course. Although GAMEFULQUEST has been psychometrically validated, we decided to do an EFA based on the fact that the instrument is new, and the three services used in the validation study were either standalone videogames (Zombies, Run!) or purposefully gamified systems (Duolingo and Nike+ Run Club). The validated 56-item GAMEFULQUEST inventory consists of seven dimensions: Accomplishment, Challenge, Competition, Guided, Immersion, Playfulness, and Social Experience (Högberg et al., 2019). Since our study focuses on the viewpoint of an autonomous learner and her motives, we decided to develop a total of eight new items for GAMEFULQUEST for measuring experienced autonomy as a part of a gameful experience. Similarly, to our approach with the MAL inventory, we assessed sampling adequacy for GAMEFULQUEST both in the validated form and as complemented by eight items that supposedly measured experienced autonomy while studying on the online course. Both the validated GAMEFULQUEST (*KMO* = 0.975, the Bartlett test of sphericity: *chi-square =* 27,407, *df* = 1,596, *p* = 0.000) and the version complemented by eight autonomy-based items (*KMO* = 0.978, Bartlett test of sphericity: *chi-square =* 31,083, *df* = 2016, *p* = 0.000) clearly passed the tests.

Scales for measuring student engagement are often developed for a school context thus necessitating modifications to any chosen instrument when applied to online learning. Engagement with the online course was studied using the Student Engagement Inventory (Lam et al., 2014). The 33-item scale explores three dimensions of engagement: Affective Engagement (nine items), Behavioral Engagement (12 items), and Cognitive Engagement (12 items). A number of the items were explicitly designed for a school context, thus, for the purposes of this study several items were excluded from our analyses on how motives to participate may predict engagement and whether experienced gamefulness and experienced engagement are correlated. The number of items was reduced to four items per dimension, and the resulting 12-item inventory Cronbach’s alphas (95% confidence intervals for the alphas) and coefficient omegas were for each dimension: *Affective Engagement α =* 0.872 *(CI 0.93–0.95)* and *ω =* 0.882, *Behavioral Engagement α =* 0.693 *(CI 0.65–0.73)* and *ω =* 0.7326, and *Cognitive Engagement α =* 0.8263 (*CI 0.80–0.85*) and *ω =* 0.8321.

According to Ames (1992, p. 267–268), in studying motivational climates it is essential to look into the learner’s perceptions of learning experiences rather than mere learner behavior. To measure participants’ perceptions of how the online course supports their motivation through different instructional strategies we used the 18-item version of the Motivating Instructional Contexts Inventory (MICI) validated (Cronbach’s *α* = 0.94) by Lam and Law (2007). The scale consists of six 3-item subscales: Challenge, Real-Life Significance, Curiosity, Autonomy, Recognition, and Evaluation. As the scale was developed for teacher-led classroom learning contexts, two subscales (Recognition and Evaluation) not suited to the online course studied here were excluded, and the remaining items were reworded to apply to a learning context where a teacher is not present. Furthermore, the four subscales used in the correlation analyses of this study were renamed to Challenge Support, Application Support, Curiosity Support, and Autonomy Support to better suit an online learning context. Cronbach’s *α* and McDonald’s *ω* were calculated for each subscale: Challenge Support *ω* = 0.69 (*CI* 0.65 to 0.73), ω = 0.71, Curiosity Support *α* = 0.64 (*CI* 0.59 to 0.68) and *ω* = 0.64, Application Support *α* = 0.857 (*CI* 0.84 to 0.87) and *ω* = 0.861, and Autonomy Support *α* = 0.70 (*CI* 0.66 to 0.74) and *ω* = 0.71.

Finally, we needed to measure participants’ perceptions of their learning outcomes. For this purpose we developed three items for assessing participants’ perceptions of learning about the course content (Learning the Topic, *α* = 0.762, *CI* 0.73 to 0.79, *ω* = 0.795), and three items to evaluate their perceptions of learning methods for online learning (Learning Method, *α* = 0.889, *CI* 0.87 to 0.90, *ω* = 0.892). Additionally, we used the 4-item Critical Reflection scale (Kember et al., 2000) to gauge the learning outcomes of critical thinking skills (*α* = 0.836, *CI* 0.81 to 0.86, *ω* = 0.837). A sum variable for each of these measures was calculated for investigating learning outcomes by means of multiple linear regressions between the above-mentioned constructs.

### Participants and Data Collection

Data collection was conducted in two languages, English and Finnish. The survey was translated from English to Finnish using the committee approach. First, the survey was translated by the researchers, who are all competent in both languages. Next, a back-translation from Finnish to English was done by an outside translator. Then the back-translation was used to assess the original translation as well as the wording in the new instruments developed for this survey.

Survey participants were recruited through the Elements of AI course email list with the message targeted at students from Finland, Germany, Sweden, and the United Kingdom. To exclude respondents who had only recently registered on the course, the survey was further targeted at participants who had registered at least 6 months ago. The participants had the opportunity to participate in a raffle for a 200€ gift card. The resulting sample included 705 responses, with 54% of the respondents reporting they were female, 44.5% male, and 0.3% other, while 1.1% did not want to specify. The mean age of the sample was 47.8 (*Min* = 16, *Max* = 83). Three of the participants were minors. The Finnish national board on research integrity states that a person’s own consent for participation is sufficient when the participant is 15 or older. As our data gathering method was anonymous, we did not have a chance to inform the parents of participating minors. All participants provided their written informed consent to participate in this study.

For the present study, it is important to note that as many as 82% of the respondents answered that they participated in the course out of their own interest. Only 10% of the respondents participated in the course as a part of workplace training, and 8% as a part of degree education. This observation is in line with our expectation that participation in MOOCs should be voluntarily initiated. And it indeed implies that the great majority of the survey respondents represent autonomous learners.

## Results

### MAL Inventory

Next, an exploratory factor analysis for the MAL inventory was made. The purpose of making an EFA was to analyze whether the MAL inventory measured latent motive dimensions, and to what extent these possible latent motive dimensions could be considered to be context-specific and to what extent, consequently, reoccurring across multiple contexts of autonomous experiencing.

We conducted a parallel analysis (Henson and Roberts, 2006) to identify how many factors should be extracted for the 35-item MAL inventory (Table 1). Both the parallel analysis (PA) with 100 iterations as well as the Kaiser eigenvalue >1 rule suggested that eight factors were to be extracted, and therefore we proceeded to investigate an eight-factor solution. Promax rotation was selected over orthogonal rotation methods, such as varimax rotation, since it is reasonable to assume that motives to participate are correlated to each other in all real-life situations (see Matsunaga, 2010, p. 100). We used factor loadings over 0.4 as a criterion of whether an inventory item had loaded on a factor (Hair et al., 2010), and considered three items to be the sufficient minimum number of items for a factor to be identified (Brown, 2015).

In scale validation studies, it is recommended to first remove items that do not meet the selected inclusion criteria, and then investigate again how many factors should be extracted for the inventory under investigation (Matsunaga, 2010). However, the objective of our study was not to validate the MAL inventory but rather to explore how all of the included motive items behaved in relation to each other. Therefore, we decided not to exclude items that showed loadings under 0.4 for all factors. Instead, we focused only on the latent dimensions and whether at least three items showed a loading over 0.4 on each of them.

In the eight-factor solution, no items loaded on the eighth factor with a loading over 0.4. Therefore we decided to extract seven factors. In the seven-factor solution, only one item “because of the certificate” loaded to the seventh factor with a loading over 0.4. Since the seventh factor was not properly identified, we rejected this solution and extracted a six-factor solution. In the six-factor solution, at least three items loaded on each factor with a loading that exceeded 0.4. This solution was thus retained, and we report the full factor loading table below (Table 2).

**Table 2:** Full factor loading table and uniqueness for the 35 Motives for Autonomous Learning inventory.

Item	Capability	Autotelic	Achievement	Social	Boredom	Reward	Uniqn.
motive19	**0.85**	−0.19	−0.05	0.00	0.01	0.13	0.41
motive21	**0.83**	−0.17	−0.01	−0.04	0.05	0.08	0.44
motive22	**0.75**	0.00	−0.01	0.01	0.07	−0.15	0.44
motive17	**0.62**	0.19	−0.09	−0.03	−0.05	−0.07	0.49
motive14	**0.60**	0.08	0.14	−0.08	−0.06	−0.04	0.48
motive15	**0.57**	0.12	−0.01	−0.03	0.01	−0.08	0.59
motive12	**0.54**	0.09	0.19	−0.14	−0.09	0.04	0.52
motive30	**0.48**	0.05	−0.11	0.15	0.00	0.14	0.70
motive27	0.36	0.05	0.20	0.15	0.04	0.02	0.71
motive18	0.34	0.33	0.07	−0.08	0.00	−0.26	0.54
motive23	−0.06	**0.74**	−0.12	0.05	−0.05	0.02	0.54
motive25	0.06	**0.66**	−0.06	0.08	0.01	0.15	0.50
motive24	−0.10	**0.55**	−0.10	−0.03	0.34	0.01	0.55
motive13	0.22	**0.45**	−0.07	0.09	−0.03	0.10	0.65
motive32	0.35	0.37	0.05	−0.11	−0.08	−0.19	0.51
motive16	0.29	0.35	0.10	0.01	0.08	−0.08	0.63
motive6	0.15	0.33	−0.01	−0.13	−0.11	0.32	0.75
motive34	0.02	−0.14	**0.80**	0.01	0.04	0.10	0.39
motive11	0.14	−0.17	**0.70**	0.12	−0.03	−0.04	0.44
motive7	0.22	−0.01	**0.61**	0.03	−0.09	0.12	0.46
motive33	−0.09	0.10	**0.61**	0.04	0.04	−0.04	0.61
motive4	−0.11	0.23	**0.53**	0.05	0.09	0.02	0.61
motive28	−0.11	0.07	0.03	**0.80**	−0.11	−0.07	0.40
motive31	−0.05	0.01	0.06	**0.79**	−0.09	−0.01	0.39
motive29	−0.04	0.01	−0.02	**0.69**	0.05	−0.09	0.55
motive9	0.04	0.02	−0.01	−0.05	**0.81**	−0.04	0.38
motive10	0.00	−0.03	−0.03	−0.09	**0.80**	0.00	0.38
motive8	−0.04	0.02	0.08	−0.06	**0.74**	0.02	0.45
motive2	−0.05	0.05	**0.49**	−0.10	0.00	**0.61**	0.44
motive1	−0.25	0.03	**0.50**	−0.02	−0.07	**0.45**	0.60
motive3	0.02	−0.06	**0.65**	−0.06	0.03	**0.40**	0.46
motive5	0.00	0.17	0.02	−0.07	0.03	0.35	0.85
motive20	0.26	−0.02	0.07	0.25	0.14	0.22	0.71
motive26	0.08	−0.02	0.02	0.23	0.13	0.13	0.87
motive35	0.00	0.22	0.02	0.23	0.13	0.05	0.83
Mean (sum)	5.721	4.714	3.741	2.874	2.352	3.313	
Std.	0.844	1.245	1.367	1.593	1.407	1.583	
Cronbach’s Alpha	0.858	0.725	0.8077	0.812	0.836	0.753	
McDonald’s Omega	0.868	0.732	0.8116	0.817	0.837	0.762	

In the exploratory factor analysis eight motive items loaded on the first factor (Table 2). These items describe motives to participate, because an individual wants to keep up with the times and stay ahead of technological changes and innovations. A person motivated by this factor is curious about what the future might bring and participates in the course to explore new domains of knowledge which build her skills and appear to be useful in the future. We call this motive dimension *Capability* and it clearly refers to the value that participating in the course might bring for the individual in the future. This motive factor was also the most important reason for participating in the online course (mean 5.72).

The second factor indicates a motive for participating, because the learning experience is expected to be fun, challenging, and entertaining. Furthermore, a person motivated by this factor also took the course because participating made them feel good about themselves. We can also explore those items that showed loadings over 0.3 but under 0.4 on this factor and conclude that this motive dimension is also associated with curiosity, creativity, and interest in the subject matter as well as the fact that the person can freely choose when and how she participates in the course. Generally speaking, these motive items refer primarily to the *learning experience itself* instead of its possible consequences and future applications similarly to the *Capability* factor. Moreover, the items that loaded on this factor are similar to how intrinsically motivating and especially playful experiences have been described in the literature (Ryan and Deci, 2000; Sansone and Harackiewicz, 2000; Rheinberg and Engeser, 2018). Finally, the items that showed lower loadings on this factor concern the participant’s ability to act freely and autonomously. We call this motive factor *Autotelic* and this concept designates that the activity has a purpose in itself and that the learning experience is what draws us to participate in the course. *Autotelic* had the second highest mean value (4.71) and it can therefore be considered as the second most important motive to participate in the Elements of AI course.

Eight motive items loaded on the third factor. Three of these items cross-loaded between this factor and the sixth factor, but five items showed high loadings only on the third factor. These latter motive items describe reasons to participate in the course because it was considered useful for career development and professional development, because it was expected to open up new future possibilities, and because it was anticipated that participating would help to chase dreams. Furthermore, the items that cross-loaded between this factor and the sixth factor denote participating because of the certificate, the course credits, and because participating would be beneficial for building up one’s resumé. All of the above-mentioned items portray the *Achievement* motive factor which had the third highest mean value (3.74).

Three items loaded on the fourth factor. These motives indicate that an individual participates because the course was recommended to her, because she was encouraged to take the course, and because her friends or colleagues also took the course. We label this factor *Social* and it had the second lowest mean sum of the six factors (2.87).

Three items also loaded on the fifth factor. These items describe that a person participated in the course because she had too much free time, because she had nothing else to do, and because she was bored. Consequently, we call this motive factor *Boredom* and it was the least important reason for participating in the online learning course (mean 2.35).

The sixth factor also had three items that loaded on it. All of these three items showed a loading also on the *Achievement* factor. These items denote short-term prizes and *Reward*, and only one item “Because of the certificate.” The *Reward* factor was the fourth most important reason to participate in the course (mean 3.31).

Finally, we should also consider those items that did not show loadings over 0.4 on any of the six identified factors. As mentioned earlier, the items that described interest toward the course subject, curiosity, and autonomy all showed loadings over 0.3 on *Autotelic*. In addition to that, the items describing interest toward the subject matter also showed loadings over 0.3 on *Capability,* whereas the item “because I could study anywhere and anytime” cross-loaded between *Autotelic* and *Boredom*. The motive item “…because it was free” did not load on *Autotelic* but it did show a loading over 0.3 on *Boredom*. Finally, the three items which denoted fear of missing out, fear toward AI or robots, and desire to evaluate the course quality did not show loadings over 0.25 on any of the six factors.

The EFA reported in Table 2 suggests that motives for autonomous learning can be identified. The motives *Capability*, *Autotelic*, *Achievement*, *Social*, *Boredom*, and *Reward* reflect participants’ desire for learning, but are not otherwise drastically different from motives to play games (H1). Psychometric studies on motives for gameplay frequently report that social interaction, boredom, achievement, and especially qualities inherent to the experience itself (e.g., fun and challenge), are prevalent reasons to engage with games (Sherry et al., 2006; Yee, 2006; Demetrovics et al., 2011; Yee et al., 2012; Kahn et al., 2015; De Grove et al., 2017; Vahlo and Hamari, 2019). However, it is much rarer that a validated measure that assesses motives to play games would include dimensions that denote only short-term rewards and prizes (*Reward*) or players’ desire to build their skills and capabilities that would extend to their career development or their future prospects (*Capability*).

### Gamefulness of the Contextual Engagement

Next, we continued to examine the second hypothesis of this study: “*H2: Gameful qualities are not exclusive to games and gamified solutions but instead these qualities are manifested across all learning situations.*” For this purpose, we made another exploratory factor analysis (EFA), this time for the GAMEFULQUEST measure. We investigated the number of factors in the 64-GAMEFULQUEST inventory by doing a parallel analysis test with 100 iterations. The PA test as well as Kaiser eigenvalue >1 test suggested eight factors for the 64-GAMEFULQUEST. Since GAMEFULQUEST is a validated measure, we utilized factor loading over 0.40 (promax rotation) as a criterion to determine whether an inventory item loaded on a factor (Hair et al., 2010). In the first iteration, no items loaded on the eighth factor of the 64-GAMEFULQUEST. After excluding a total of seven items from the original 56-GAMEFULQUEST and one of the additional autonomy-based items (“Was a chance for me to fulfill myself and live by my values”) which did not load on any factor, we made another PA test for the inventory. The test suggested a 6-factor solution. In the second iteration, one more item did not load on any of the factors. The PA test still suggested that six factors should be extracted from the inventory, and in the third iteration all remaining items loaded on a factor, and at least three items loaded on each factor. The full factor loadings are reported in Table 3.

**Table 3:** Factor loading table and uniqueness for GAMEFULQUEST, complemented by autonomy-based items (marked with “^*^” in the table).

Description	Purposeful Play	Social Connectedness	Guided	Competition	Absorption	Effort	Uniqn.
Let me work on my own terms^*^	**0.79**	0.02	−0.15	−0.07	−0.07	−0.19	0.59
Encouraged me to strive for achievements	**0.74**	−0.10	0.06	0.11	−0.07	0.05	0.42
Motivated me to improve and become better	**0.73**	0.00	0.07	0.04	−0.05	0.06	0.38
Inspired me to maintain my performance level	**0.73**	−0.04	−0.11	0.06	0.08	0.04	0.44
Gave me the experience of being able to participate on my own terms^*^	**0.72**	0.04	−0.06	−0.06	−0.03	−0.14	0.59
Made me feel that I am finding new things	**0.70**	−0.04	0.03	−0.17	0.08	0.14	0.45
Made me pursue the next level	**0.69**	−0.04	0.05	0.05	0.01	0.11	0.40
Made me feel that I want to know what happens next	**0.66**	−0.02	0.10	−0.04	0.05	−0.02	0.50
Made me feel that I was exploring things	**0.63**	−0.04	0.04	−0.07	0.16	0.09	0.45
Made me feel that I had bring things to conclusion.	**0.63**	−0.08	0.09	0.14	−0.13	0.05	0.56
Made me feel that I have clear goals	**0.61**	0.04	0.07	0.13	−0.04	0.00	0.49
Made me feel that I had to achieve goals.	**0.60**	−0.09	0.08	0.20	−0.10	0.14	0.49
Appealed to my curiosity	**0.59**	0.01	−0.06	−0.10	0.18	−0.06	0.62
Gave me the freedom to make my own choices^*^	**0.59**	0.11	0.09	0.03	−0.05	−0.17	0.59
Made me feel that I know what I have to do in order to advance	**0.56**	0.12	−0.01	0.07	0.04	0.05	0.50
Made me feel that I was free to make my own choices^*^	**0.52**	0.15	0.16	0.01	−0.02	−0.16	0.58
Let my imagination run wild	**0.52**	0.08	0.03	−0.09	0.26	−0.03	0.51
Made me feel that my own activity and participation were important^*^	**0.51**	0.19	−0.01	−0.03	−0.04	0.14	0.54
Was a chance for me to fulfill myself and live by my values^*^	**0.51**	0.18	−0.10	0.11	0.08	0.00	0.53
Showed me that the choices I made matter.^*^	**0.48**	0.15	0.17	0.07	−0.07	0.01	0.51
Gave me useful feedback so that I can adapt	**0.45**	0.21	0.19	−0.06	−0.06	−0.01	0.55
Made me feel creative	**0.43**	0.12	0.10	0.08	0.19	−0.01	0.46
Made me feel that in order to succeed I have to constantly improve	**0.43**	0.01	−0.01	0.12	−0.03	0.30	0.55
Pressured me to reach for higher goals, in a positive way	**0.41**	0.06	0.01	0.07	0.02	0.32	0.50
Made me feel that I am a part of the community	0.03	**0.87**	−0.07	−0.02	−0.06	0.07	0.29
Felt like a communal experience	0.01	**0.84**	−0.04	0.04	−0.03	0.01	0.31
Made me feel that I am connected to others	0.01	**0.79**	−0.02	0.04	0.05	0.01	0.30
Affected me *via* its communality.	−0.11	**0.73**	0.05	0.08	0.01	0.09	0.37
Made me feel that I get support from the community	0.03	**0.69**	0.12	0.07	−0.01	−0.03	0.35
Made me feel that I am not alone	−0.03	**0.61**	0.22	0.00	0.06	−0.01	0.41
Made me feel that I have someone with whom to share my endeavors	0.02	**0.51**	0.21	0.09	0.08	−0.02	0.43
Made me feel that I was being guided.	0.02	0.09	**0.71**	0.02	−0.01	−0.02	0.41
Made me feel that I received guidance	0.07	0.26	**0.65**	−0.07	−0.07	0.01	0.34
Made me feel that I have a tutor	0.09	0.23	**0.59**	−0.01	0.03	0.00	0.33
Made me feel that someone kept me on the right track	0.11	0.16	**0.57**	−0.06	0.06	0.02	0.43
Made me feel that I get support in order to be organized	0.19	0.24	**0.40**	0.01	0.00	0.04	0.45
Felt like I was taking part in a competition	−0.15	0.12	−0.07	**0.79**	0.04	−0.01	0.38
Made me feel like I was in a competition	−0.11	0.12	−0.10	**0.76**	0.04	0.05	0.39
Made me want to be the number one	0.09	−0.05	0.06	**0.75**	0.01	−0.10	0.43
Inspired me to compete	0.04	−0.02	0.07	**0.73**	0.07	−0.03	0.36
Made me feel that I had to win in order to succeed	0.14	−0.02	−0.04	**0.71**	0.01	0.04	0.41
Made winning feel important	0.17	−0.05	−0.01	**0.63**	0.03	0.09	0.43
Made me participate with its competitiveness	0.07	0.17	−0.05	**0.63**	−0.03	0.02	0.45
Made me forget my everyday worries	0.05	0.09	−0.07	0.02	**0.70**	−0.03	0.45
Made me ignore everything around me	−0.05	−0.07	0.04	0.15	**0.68**	0.01	0.47
Made me forget my tiredness	0.08	0.12	0.01	0.11	**0.55**	−0.10	0.51
Took all my attention	−0.11	−0.12	0.32	0.12	**0.49**	0.20	0.45
Made me immerse myself fully in what I was doing.	**0.40**	0.01	−0.14	−0.01	**0.49**	0.00	0.50
Made me feel that time went by quickly	0.24	−0.01	0.05	−0.02	**0.48**	0.01	0.58
Demanded great effort if I wanted to succeed	−0.11	0.00	0.02	0.08	−0.09	**0.77**	0.44
Made me work at the edge of my abilities	−0.02	0.00	0.11	0.07	0.01	**0.70**	0.40
Made me test my limits	0.25	0.06	−0.08	−0.03	0.04	**0.60**	0.44
Drove me almost to the verge of giving up, in a positive way	−0.07	0.13	0.03	0.03	0.06	**0.59**	0.55
Motivated me to do things that felt very hard	0.35	0.05	0.01	−0.08	0.04	**0.56**	0.37
Challenged me	**0.44**	0.01	−0.17	−0.16	−0.02	**0.55**	0.49
Mean (sum)	4.617	3.304	3.636	2.885	3.521	3.873	
Std.	1.088	1.342	1.352	1.300	1.260	1.284	
Cronbach’s Alpha	0.958	0.929	0.888	0.912	0.847	0.866	
McDonald’s Omega	0.958	0.929	0.888	0.912	0.849	0.867	

Factor loadings over 0.4 are bolded.

The first factor consists of items that measure *Accomplishment* (e.g., “Encouraged me to strive for achievements”) and *Playful* (“Made me feel that I am finding new things”) in particular, but also to some extent *Challenge* (e.g., “Made me feel that in order to succeed I have to constantly improve”) and *Guided* (e.g., “Gave me useful feedback so that I can adapt”) in the validated 56-GAMEFULQUEST. In addition, seven of the eight items we developed for measuring experienced autonomy loaded on this factor. More precisely, the items that showed the highest loadings on the first factor were those that we developed for measuring experienced autonomy and those items that measure *Accomplishment* in the validated 56-GAMEFULQUEST. Since the first factor included items from four factors of the validated 56-GAMEFULQUEST as well as the new autonomy-based items, the factor could not be labeled similarly to any of the GAMEFULQUEST dimensions. We decided to call this factor *Purposeful Play*. The word “purposeful” refers to the experienced autonomy, and accomplishments that result in overcoming challenges in a feedback-providing environment. In addition to the clear reference to playfulness, the word “play” also refers to autonomy as play is frequently described as a voluntary act of expressing oneself freely.

The second factor consisted of the same items as a factor in the validated GAMEFULQUEST, and therefore we similarly call it *Social Experience*. The items that loaded on the third factor and the fourth factor were the same that loaded on the *Guided* and *Competition* in the validated GAMEFULQUEST, and we decided to retain these factor names. The fifth factor was very similar to the *Immersion* factor in the 56-GAMEFULQUEST, but the item “Made me immerse myself fully in what I was doing” cross-loaded with *Purposeful Play*. Since this item refers directly to immersion, we re-considered the name of the fifth factor and decided to call it *Absorption* instead of immersion. Finally, the sixth factor was similar to the *Challenge* factor of the GAMEFULQUEST, but again the item that includes the word “challenge” cross-loaded with *Purposeful Play*. Hence, we call this factor *Effort*, as the items that have the highest loading on this factor describe the work required from the participant more precisely than the challenges the course imposes on them.

In addition to the results of the EFA reported in Table 3, we also investigated whether a similar six-factor structure would be present in the validated 56-GAMEFULQUEST without the autonomy-based items fashioned by our research group. Applying the same EFA procedure as described above with the 64-GAMEFULQUEST resulted in a six-factor solution instead of an anticipated seven-factor solution (Högberg et al., 2019). In the six-factor solution, items that measure *Accomplishment* and *Playful* in the original GAMEFULQUEST still loaded on the same factor alongside with the item “Challenged me” from *Challenge* and “Gave me useful feedback so that I can adapt” from *Guided*. The other five factors were similar to the ones identified with the 64-GAMEFULQUEST and reported in Table 3.

The GAMEFULQUEST inventory did not measure the seven dimensions similar to the scale validation study (Högberg et al., 2019). Regardless of this, the inventory did measure gameful dimensions of the online course experience, which provides some support for the second hypothesis. By observing the mean sums for each of the identified factors, we can conclude that the experienced gameful qualities of participating in the online learning consisted mostly of *Purposeful Play* (mean 4.6) and *Effort* (mean 3.9) whereas *Competition* had a clearly lower mean value (2.9) than any of the other dimensions.

Next, we investigated how experiences of instructional practices of the learning situation were related to participants’ explicit motives for taking the course (Table 2) and their experienced gamefulness of the learning situation (Table 3). This was done by calculating correlations between sum variables of the four dimensions of the MICI and (1) the motives to participate in the course (MAL), as well as (2) dimensions of the experienced gamefulness of the situation (GAMEFULQUEST). The results of these correlations are reported in Table 4.

**Table 4:** Correlations (Spearman’s Rho) between the four Motivating Instructional Contexts Inventory (MICI) factors, and dimensions of experienced gamefulness and motives to participate in the course.

	Real-Life Significance Support	Autonomy Support	Curiosity Support	Challenge Support
***Motivational and Instructional Inventory***
Real-Life Significance Support	1.00			
Autonomy Support	**0.43**	1.00		
Curiosity Support	**0.64**	**0.49**	1.00	
Challenge Support	**0.61**	**0.43**	**0.56**	1.00
***Gamefulness of the learning experience***
Purposeful Play	**0.63**	**0.55**	**0.59**	**0.57**
Social Connectedness	0.29	**0.48**	0.35	0.29
Guided	0.26	**0.40**	0.30	0.19
Competition	0.06	0.30	0.18	0.05
Absorption	0.28	0.38	0.35	0.31
Effort	0.25	0.27	0.36	0.13
***Motives to participate***
Capability	**0.50**	0.29	0.37	0.33
Autotelic	**0.40**	0.38	0.35	0.37
Achievement	0.25	0.19	0.21	0.07
Social	−0.04	0.10	0.11	−0.00
Boredom	−0.15	0.04	−0.06	−0.04
Reward	−0.03	0.00	0.01	0.07

Correlations over 0.4 are bolded.

Experienced gamefulness in the form of *Purposeful Play* was found to be strongly correlated with all of the dimensions of the MICI. Experienced *Social Connectedness* and *Guided* of the GAMEFULQUEST were both correlated with *Autonomy Support*. In addition to these correlations, several dimensions of experienced gamefulness were weakly correlated with MICI dimensions. The motives of *Capability* and *Autotelic* were the only two participation reasons that were clearly associated with the MICI dimensions, and these correlations were the strongest between *Real-Life Support* and *Capability,* and *Real-Life Support* and *Autotelic*.

### Engagement in the Learning Situation

We proceeded to calculate factor score variables for both MAL factors (Table 2) and GAMEFULQUEST dimensions (Table 3). We decided to use factor score variables in the following analyses instead of factor sums as factor scores provide information about how an item loads on every factor (DiStefano et al., 2009). Calculating factor scores is possible after making an EFA, and it was a reasonable approach in our situation in which we wanted to retain all inventory items and also items that showed cross-loadings between several factors (Tables 2 and 3). In order to investigate whether motives for autonomous learning (H3a) and perceived gamefulness of the learning experience (H3b) were associated with experienced engagement, we calculated factor sums for the Student Engagement Inventory (Lam et al., 2014).

Since the survey respondents were asked in the questionnaire to specify the reasons, that is, explicit motives, why they decided to take the online course, it is plausible to assume that motive factors are possible precedents for experienced engagement in a learning situation. To analyze the relationship between the motives (Table 2) and experienced engagement, we first calculated a linear regression between a combined motive sum variable and combined engagement sum variable. Both of these variables were constructed over all items in their corresponding inventories. The linear regression between the motive sum variable and the engagement sum variable showed that the motives combined do predict experienced engagement (coefficient 0.49, standardized error 0.04, *p* = 0.000, *t* = 12.87, *β* = 0.44, *R2* = 19%). Next, we calculated multiple linear regressions between the identified motive factors and *Affective engagement, Behavioral engagement, and Cognitive engagement.* The results of these regressions are presented in Table 5.

**Table 5:** Multiple regressions between autonomous motives to participate in a learning experience and experienced level of engagement.

Motives	Affective		Behavioral		Cognitive	
	*Beta*	*Std. Err*	*Beta*	*Std. Err*	*Beta*	*Std. Err*
Capability	***0.18***	0.04	***0.19***	0.04	***0.30***	0.04
Autotelic	***0.50***	0.04	***0.36***	0.05	***0.27***	0.05
Achievement	−0.01	0.03	***0.11***	0.04	0.05	0.04
Social	**−0.08**	0.04	−0.05	0.04	−0.03	0.04
Boredom	***–0.23***	0.04	***–0.20***	0.04	***–0.16***	0.04
Reward	0.05	0.04	***0.19***	0.04	−0.02	0.04
***R* ^2^**	***0.38***		***0.31***		***0.30***	

*p* < 0.05 are bolded, *p* < 0.01 are bolded and italicized, and *p* < 0.001 are bolded, italicized, and underlined.

Both *Capability* and *Autotelic* motives to participate predicted all three aspects of engagement. The effect of *Capability* on engagement was largest on *Cognitive engagement* whereas *Autotelic* was the main precedent for both *Behavioral engagement* and especially *Affective engagement*. *Achievement* and *Reward* motives were associated with *Behavioral engagement* whereas *Social* motives predicted lower *Affective engagement*, albeit weakly. Instead, the *Boredom* motive predicted a lower level of engagement from the perspective of all three engagement dimensions. A hypothesis of this study (*H3a*) was that all user motives would predict a higher level of engagement. If all motive factors are combined, the effect of motives on engagement is positive. But when each motive dimension is considered individually, it is revealed that not all motives are associated with a deeper level of engagement. On the contrary, especially participating out of *Boredom* was found to predict a significantly lower level of affective, behavioral, and cognitive engagement in a learning experience. Therefore, this hypothesis (*H3a*) of this study was not supported.

It was also hypothesized that experienced gamefulness of a situation would be associated with engagement. Since both perceived gamefulness and engagement are part of the learning situation and how a user experiences the motivational climate, it is reasonable to assume that experienced gamefulness would predict engagement or that engagement would be a precedent for experienced gamefulness. Therefore, we calculated correlations (Spearman’s Rho) between these two constructs to investigate their possible linkages (Table 6).

**Table 6:** Correlations (Spearman’s Rho) between experienced gamefulness and dimensions of engagement in a learning experience.

	Purposeful Play	Social Connectedness	Guided	Competition	Absorption	Effort
Affective Engagement	**0.68**	0.27	0.28	0.10	0.34	0.25
Behavioral Engagement	**0.64**	0.31	0.31	0.28	0.37	**0.41**
Cognitive Engagement	**0.58**	0.22	0.17	0.10	0.25	0.21

Moderate and stronger correlations (over 0.4) are bolded.

Experienced *Purposeful Play* in a learning situation was strongly correlated with all of the three modes of engagement, most strongly with *Affective engagement* and *Behavioral engagement*. Of the other five dimensions of gameful experience, only *Effort* was moderately correlated with one dimension of engagement, *Behavioral engagement*. The other four dimensions of gameful experience had only weak or very weak linkages with engagement dimensions. These results did not provide support for hypothesis H3b: “*Gameful qualities of a learning situation are associated with engagement.*”

### Identifying Linkages Between Motives, Gamefulness, and Learning Outcomes

Following Masek and Stenros (2021), we have approached playfulness in a two-fold fashion: playfulness is the *desire and intent for playful engagement* and the *playful organization of an experience.* Similarly to Janosz (2012), we have also stated that participants’ motives are not to be understood as isolated from the experience, but rather as meta-regulative practices that participants engage with before, during, and after an activity. Following these demarcations, we hypothesized that gameful qualities of a learning situation are not identical between participants but are instead influenced by their motives to participate and organize the situation playfully (H4). In an effort to study this hypothesis, we calculated multiple regressions between MAL factor score variables and GAMEFULQUEST factor score variables.

All six motive dimensions were found to be associated with at least one dimension of GAMEFULQUEST. Experienced *Purposeful Play* was strongly predicted by the *Autotelic* motive to participate, but also by *Capability, Achievement*, and *Reward*. However, the *Boredom* motive predicted a lower level of experienced *Purposeful Play* in a learning situation. *Social Connectedness* was predicted by *Autotelic* and *Social* motives, but also by *Achievement* albeit only weakly. Experienced *Guided* was predicted by *Achievement*, *Autotelic*, and *Social.* Similarly, *Competition* was also predicted by *Achievement* and *Social*, but also by *Boredom*. *Absorption* was strongly predicted by *Autotelic*, and *Capability* and *Social* were both precedents for experienced *Effort*.

The motive dimensions explained 48 percent of the variance of *Purpose Play* and also over 20 percent of all other GAMEFULQUEST factors. The multiple regressions (Table 7) therefore suggest that gameful qualities of a learning situation are co-constituted by both the characteristics of the learning environment and by the participants motives to participate and organize the situation playfully. This supports the fourth hypothesis of this study.

**Table 7:** Multiple regressions between MAL factor scores and GAMEFULQUEST factor scores.

Motives	Purposeful Play		Social Connectedness		Guided		Competition		Absorption		Effort	
	Beta	Std. Err	Beta	Std. Err	Beta	Std. Err	Beta	Std. Err	Beta	Std. Err	Beta	Std. Err
Capability	***0.20***	0.04	**0.11**	0.04	0.02	0.04	**0.09**	0.05	−0.01	0.04	***0.29***	0.04
Autotelic	***0.50***	0.04	***0.25***	0.05	***0.26***	0.05	0.08	0.05	***0.48***	0.04	0.08	0.05
Achievement	***0.11***	0.04	***0.16***	0.04	***0.27***	0.04	***0.26***	0.04	0.06	0.04	0.04	0.04
Social	0.03	0.04	***0.22***	0.04	***0.15***	0.04	***0.18***	0.04	**0.09**	0.04	***0.20***	0.04
Boredom	***–0.13***	0.03	**0.08**	0.04	0.07	0.04	***0.20***	0.04	**0.10**	0.04	0.02	0.04
Reward	**0.10**	0.04	−0.03	0.05	−0.06	0.04	0.03	0.05	−0.02	0.04	0.08	0.05
***R* ^2^**	***0.48***		***0.27***		***0.26***		***0.25***		***0.31***		***0.21***	

*p* < 0.05 are bolded, *p* < 0.01 are bolded and italicized, and *p* < 0.001 are bolded, italicized, and underlined.

The final hypotheses we investigate consider how explicit motives and experienced gamefulness influence learning outcomes. Based on how prior research has argued that motives are both determinants for engagement and learning (Janosz, 2012) and that gameful experiences also have a positive effect on learning outcomes (Hamari et al., 2016), we hypothesized that: *H5a: Gameful experience is a precedent for learning outcomes* and that *H5b: Explicit motives are associated with learning outcomes via gameful experience.*

The effects of MAL motive dimensions and GAMEFULQUEST categories were investigated by utilizing structural equation modelling (CB-SEM) and by constructing the model presented in Figure 1. The statistical analyses were made by statistical software Stata 16.2 using the maximum likelihood method. The variables in the model are all factor score variables with the exception of the outcome variables, all of which are sum variables.

**Figure 2:**
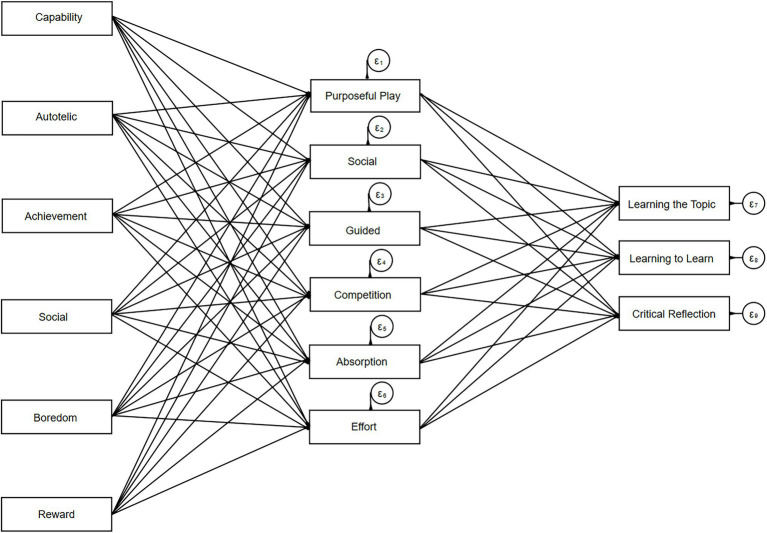
Structural model for investigating the direct effects of experienced gamefulness and indirect effects of explicit motives on learning outcomes.

The model described in Figure 2 and reported in Table 8 explained 19 percent of the variance in *Learning Method*, 31 percent of the variance in *Learning the Topic,* and 30 percent of the variance in *Critical Reflection*. Experienced *Purposeful Play* had a strong effect on *Learning the Topic*, and also the strongest effect of the six GAMEFULQUEST dimensions on *Learning Method.* The factor of *Social Connectedness* had the strongest effect on *Critical Reflection*, followed by *Purposeful Play*. The only effects that *Guided* and *Competition* had on the learning outcomes were negative. Finally, the MAL motive dimensions also had statistically significant effects on all learning outcomes *via* the experienced gamefulness of the learning situation. Again, *Autotelic* had the strongest effects of the motive dimensions, but also *Capability* and *Social* had positive impacts on learning. The effect of *Achievement* on learning was very weak, the *Boredom* motive had mostly negative influence on learning, although these effects were also very weak.

**Table 8:** The direct effects of experienced gamefulness and indirect effects of motives on learning.

			*Direct Effects*			
	Learning the Topic		Learning Method		Critical Reflection	
	*Beta*	*SD*	*Beta*	*SD*	*Beta*	*SD*
Purposeful Play	***0.57***	0.04	***0.33***	0.05	***0.21***	0.05
Absorption	0.02	0.04	−0.02	0.05	***0.17***	0.06
Effort	**0.10**	0.03	***0.16***	0.04	***0.17***	0.05
Social connectedness	−0.08	0.04	**0.14**	0.05	***0.27***	0.06
Guided	−0.01	0.04	−0.06	0.05	***−0.14***	0.06
Competition	***−0.12***	0.04	−0.06	0.05	0.04	0.06
			***Indirect Effects***			
	**Learning the Topic**		**Learning Method**		**Critical Reflection**	
**Motives**	*Beta*	*SD*	*Beta*	*SD*	*Beta*	*SD*
Capability	***0.12***	0.02	***0.12***	0.02	***0.12***	0.03
Autotelic	***0.27***	0.02	***0.18***	0.03	***0.24***	0.04
Achievement	0.02	0.02	0.03	0.02	**0.06**	0.03
Social	0.00	0.02	***0.05***	0.02	***0.10***	0.03
Boredom	***−0.10***	0.02	**−0.04**	0.02	0.01	0.03
Reward	**0.06**	0.02	***0.04***	0.02	0.03	0.03

*p* < 0.05 are bolded, *p* < 0.01 are bolded and italicized, and *p* < 0.001 are bolded, italicized, and underlined.

## Discussion

Our guiding principle for this explorative study has been the idea that *learning* and *playing*, the activities that are still more or less considered through different academic contexts, carry phenomenal similarities that could be revealed by researching autonomous learners’ motives for participating in a MOOC that does not involve any intentionally gameful features. We conducted the investigation empirically by collecting survey responses from students of the free Elements of an AI online course. The collected self-report measures concerned their experiences of (1) motives to participate in the course, (2) contextual engagement during the course, and (3) learning outcomes. In regard to these domains of the learning process, we carried out three respective research tasks in which we investigated: (1) how playfulness/gamefulness was manifested in participants’ motives for learning, (2) how engagement with the course exhibited playful/gameful organization of experiences and how the measured gamefulness related to both learning motives and engagement, and finally (3) how playful/gameful motives and engagement associated with the learning outcomes.

The results of this study demonstrated that playfulness and autonomy were both prominent and significant factors across the learning process. Playfulness was here operationalized in a two-fold manner, conforming to Masek and Stenros’s (2021) conceptualization that focuses on both an individual’s playful dispositions (i.e., playful motives) and playful or gameful organization of learning experiences. *Autotelic* motives, in which the learning experience was expected to be fun, challenging, and entertaining, and which also denoted a person’s interest in experiencing autonomy, yielded the greatest (indirect) positive effect on learning outcomes, as well as the greatest effect on gameful organization of learning experiences (H5b). Of the motive categories, *Autotelic* motives most clearly highlighted the nature of a playful disposition. Moreover, several dimensions of experienced gamefulness in learning had direct positive effects on learning outcomes (H5a). An important thing to note here is that the playful and gameful dimensions that seemed to have the most significant role did not relate to short-term rewards or competition, but rather, they related to a more profound and playful predisposition that was associated with freedom of choice and autonomy. This parallels some previous notions of gamification researchers (Suh et al., 2018) asserting that gamification should strive for utilizing diverse types of gameful dynamics that help people to satisfy their long-term psychological needs. At least, our results imply that mere use of badges, points, and leaderboards would not be the best solutions for generating effective playfulness in learners. On the contrary, our results highlight that playfulness had a significant role in self-regulative motivational development during the whole process of learning: from a person’s playful attitude to the contextual engagement with learning activities that organize playful experiences and actualizes the person’s playful self.

The dimensions identified by the Motives for Autonomous Learning (MAL) inventory suggest that reasons to participate in online courses do not only inform us about learning motives but also, and perhaps even more importantly, about prevalent predispositions to engage with activities that enable and facilitate autonomous experiences. In this latter regard, it makes sense that the six factors *Capability*, *Autotelic*, *Achievement*, *Social*, *Boredom*, and *Reward* share profound similarities with motives to play digital games as participating in both of these activity types is indeed voluntary and autonomous (H1). These results indicate that models that are developed for measuring contextual motives may also generate knowledge about more general and fundamental predispositions that direct our activities across multiple contexts of experience. In game research literature, contextual and general approaches to gaming motives are often separated from each other (Ryan et al., 2006; Yee, 2006). Our research adds to this discussion by providing empirical evidence that reveals that it is not reasonable to establish a clear-cut distinction between contextual and general models, but rather to ask instead what we can learn about general human motivation by studying our reasons to engage with particular activities, such as learning and playing games.

It was revealed in this study that *GAMEFULQUEST*, an instrument developed for assessing experienced gamefulness of a situation, did not function entirely as was anticipated. The instrument did measure several dimensions similar to the model presented in the original validation study (Högberg et al., 2019), but *Playfulness* and *Accomplishment* did not form their own factors. Instead, these factors merged together with each other alongside the additional items we developed for measuring autonomy, thus forming a factor we call *Purposeful Play* (H2). The combination of those items that loaded on *Purposeful Play* reminds us of classic game definitions which argue that attainable, purposeful goals and autonomous players who are free to pursue mastery are necessary conditions for all games (Avedon, 1971; Avedon and Sutton-Smith, 1971; Mead, 2015). A reading of classic game definitions supports an interpretation of the EFA results (Table 3) according to which constitutive elements for all game experiences loaded on the *Purposeful Play* factor (autonomy, purposeful goal, challenge, and feedback) whereas frequently occurring elements of game experiences (*Social, Guided, Competition, Absorption,* and *Effort*) each formed a factor of their own. This is an important interpretation since *Purposeful Play* was revealed to be the factor that had a more significant effect on learning than the other factors of *GAMEFULQUEST.*

*Purposeful Play* did not only predict learning outcomes but this dimension was also more strongly correlated with affective, behavioral, and cognitive engagement than the other aspects of experienced gamefulness (Table 6). Again, the results revealed that not all factors of *GAMEFULQUEST* were meaningfully correlated with engagement (H3a). *Competition*, for instance, was not correlated at all with affective and cognitive engagement, and only weakly with behavioral engagement. Our investigation on how motives were associated with engagement furthermore revealed that the *Autotelic* motive was the strongest precedent for both affective and behavioral engagement whereas *Capability* and *Autotelic* both predicted a higher cognitive engagement in a learning experience. But it was found again that not all motives were positively related to engagement. The *Social* motive, for instance, was not associated with engagement at all, and the *Boredom* motive predicted a lower level of affective, behavioral, and cognitive engagement (H3b).

Furthermore, it was found that all four MICI dimensions, which relate to motivational support provided by the instructional context, strongly correlated with the *Purposeful Play* factor. Only MICI’s Autonomy Support dimension also had significant correlations to *Social* and *Guided* factors. This result implies that *Purposeful Play* exhibited a positive association with experiential dimensions of instructional support and thus arguably contributed strongly to the motivational climate of learning.

A regression between participation motives of an autonomous learner and experienced gamefulness of a learning situation revealed that motives are clearly related to how the learning situation is experienced and playfully organized by the learner. The *Autotelic* motive strongly predicted *Purposeful Play* and *Absorption*. The motive of *Capability* was associated with experienced *Effort*, and the *Achievement* motive with felt *Competition*. Again, not all motives predicted experienced gamefulness (e.g., *Reward*), and all motive factors were uniquely associated with *GAMEFULQUEST* dimensions (H4). The most striking effect revealed in this study is also the most interesting and potent. *Autotelic* was the main precedent for *Purposeful Play*, which was the most significant predictor for learning outcomes. These findings effectively reveal that experienced gamefulness is not to be regarded as a quality applicable only to games or gamified situations. Instead, aspects of gamefulness are present across learning situations, but they are also unequal from the perspective of expected learning outcomes.

### Implications

All in all, this research has shed light on understanding motives not only as *pre*dispositions for engagement (*cf.*, Thrash et al., 2012), but rather as meta-regulative resources that are immanently and dynamically involved during activities of the whole learning process. In other words, motives for participating in learning were not detached from contextual engagement. On the basis of the results, we would argue that the relationship between motives and engagement is reciprocal and co-constitutive in nature. It seems that motives are entangled with the experience of learning and, for example, how this experience is structured in terms of playfulness. As we have discussed earlier in this paper, playful motives represent only one, albeit very impactful, domain of motivational dispositions (i.e., motivation background) that has such a capacity to contribute to the organization of contextual experiences. For example, with the *Capability* motive, which particularly related to the development of one’s competence, there was a related emphasis on situationally experiencing *Effort*. Our suggestion is that future studies investigating motives and engagement should more broadly further investigate the co-constitutional nature of motives during engagement with learning activities.

In regard to practical implications, the results of this study strongly put forward an idea that playfulness or gamefulness does not have to be explicitly manifested in the design of the course material of the learning environment. As we have seen in this particular case, playfulness was exhibited as a prominent experiential aspect of participating in an online course, even though the course was not intentionally gamified. In other words, playfulness seemed to emerge as a property of the learner’s intent and the personal ways of organizing the learning experience. This aptly reminds us of Sicart’s (2014, p. 11) assertion that “play is appropriative, in that it takes the context in which it exists and cannot be totally predetermined by such context.” In practice, it means that gamification of learning, hence implementing game-like elements in the learning activities or the context, does not determine playful/gameful experiences. Gamification, rather, is about constructing the learning environments in a manner that *affords* play, that is, promotes (or at least avoids denying) the possibility of playful/gameful attitudes and experiences for the autonomous learner.

### Limitations and Future Research Directions

The present work has certain limitations that should be considered when interpreting its results and making conclusions. One of the prominent limitations relates to the fact that, instead of adopting a longitudinal methodological approach, only a single point of measurement was used for investigating different phases of the learning process. Another thing to keep in mind is that the present study focused on the users of only a single MOOC. In the future, similar research design should be replicated in studies that are targeted at different types of learning, for gaining a better understanding of the effect of different contexts in terms of different pedagogical approaches, learning topics, and motivational climates. It is also important to consider replicating the survey in a more experimental fashion, for instance in the immediate situation in which learning takes place. By doing so, the impact of motives on the ongoing experience could be investigated by making use of multi-method approaches including psycho-physiological measures and qualitative interviews. Moreover, comparative studies could also be targeted toward the users of (entertainment) game applications, and their experiences regarding motivational development.

In terms of playfulness, we may consider our study as an empirical case of Masek and Stenros’s (2021) conceptual synthesis. Hence, our findings illustrate how playfulness is reciprocally present in the motivational orientation that directs our contextual actions, as well as in the ways these contextual actions organize our experiences. The findings provide support for this conceptual framework in which playfulness is seen within two ontological stances—as an attitude and an activity—both of which are tied to contextual engagement. And finally, in terms of the basic need satisfaction of the self-determination theory (Deci and Ryan, 1991), our findings also remind us researchers not to consider basic needs, regarding autonomy, competence and relatedness, merely as background factors of motivation, but rather as meta-regulative resources for continuous motivational development.

## Data Availability Statement

The raw data supporting the conclusions of this article will be made available by the authors, without undue reservation.

## Ethics Statement

Ethical review and approval was not required for the study on human participants in accordance with the local legislation and institutional requirements. Written informed consent from the participants’ legal guardian/next of kin was not required to participate in this study in accordance with the national legislation and the institutional requirements.

## Author Contributions

JV, KT, and TV contributed to conception, design of the study, and wrote sections of the manuscript. JV organized the data and performed and reported the statistical analyses. All authors contributed to manuscript revision, read, and approved the submitted version.

## Funding

This work is funded by Business Finland, the government organization for innovation funding (9214/31/2019).

## Conflict of Interest

The authors declare that the research was conducted in the absence of any commercial or financial relationships that could be construed as a potential conflict of interest.

## Publisher’s Note

All claims expressed in this article are solely those of the authors and do not necessarily represent those of their affiliated organizations, or those of the publisher, the editors and the reviewers. Any product that may be evaluated in this article, or claim that may be made by its manufacturer, is not guaranteed or endorsed by the publisher.
